# A brief effective screening protocol for identifying cataract patients with binocular vision anomalies

**DOI:** 10.1186/s12886-024-03807-w

**Published:** 2024-12-18

**Authors:** Qing-Qing Tan, Jia-Hao Tan, Chang-Kang Luo, Chun-Yan Lai, Wei Zhao, Chang-Jun Lan, James Lewis, Saeed Aljohani, Xuan Liao, Mitchell Scheiman

**Affiliations:** 1 Department of Ophthalmology, Affiliated Hospital of North Sichuan Medical College, Nanchong, Sichuan China; 2 Medical School of Ophthalmology & Optometry, North Sichuan Medical College, Nanchong, Sichuan China; 3 Graduate Programs in Biomedicine, Salus University, Elkins Park, PA USA; 4 Chengdu Eastern Aier Eye Hospital, Chengdu, Sichuan China; 5 Pennsylvania College of Optometry at Drexel University, Elkins Park, PA USA; 6 Department of Optometry, College of Applied Medical Sciences, Qassim University, Buraydah, Qassim Saudi Arabia

**Keywords:** Binocular vision anomaly, Age-related, Cataract, Screening protocol

## Abstract

**Background:**

To compare the effectiveness of a brief binocular vision screening protocol to a comprehensive examination for detecting binocular vision anomalies before and after cataract surgery.

**Methods:**

A comprehensive binocular vision test battery as a gold standard were administered on recruited patients before the first surgery and at the third visit after surgery on the second eye. A receiver operating characteristic (ROC) curve was plotted to illustrate the diagnostic ability of each test. In addition, a univariate logistic regression analysis was performed to further determine the contribution of each preoperative test to the prediction of pre- and post-surgical binocular vision anomalies.

**Results:**

Significant differences were shown for the difference in phoria from distance to near measured by the cover test (*Area Under Curve [AUC]* = 0.96, *P* < 0.01), step vergence testing of positive fusional vergence at distance (*AUC* = 0.71, *P* < 0.01) and near (*AUC* = 0.77, *P* < 0.01). The other tests did not show statistically significant differences. The ROC curve generated by combining the difference in distance and near phoria with positive fusional vergence at both distance and near demonstrated a more robust measure of diagnostic accuracy. (*AUC* = 0.98, *P* < 0.01).

**Conclusion:**

Distance and near phoria difference measured by cover test has similar effectiveness as a comprehensive binocular vision testing protocol for the diagnosis of binocular vision anomalies. Distance and near positive fusional vergence measured by step vergence testing also have significant predictive value. A combination of the two tests is an outstanding screening protocol for binocular vision anomalies before cataract surgery.

**Trial registration:**

The study was registered at ClinicalTrials.gov (NCT03592615, Date of registration: July 19, 2018).

**Supplementary Information:**

The online version contains supplementary material available at 10.1186/s12886-024-03807-w.

## Background

The presence of cataracts is the leading cause of visual impairment across the world, and cataract treatment is in great demand [1]. Satisfactory monocular visual performance such as visual acuity and contrast sensitivity function are obtained by most patients after cataract surgery [2–4]. However a review of the literature indicates that binocular vision problems and symptoms including reading difficulty and asthenopia do occur and the reported prevalence varies from 0.093% to 6.8% [5, 6]. The prevalence of binocular vision problems before and after cataract surgery has also been observed in our previous studies [7, 8]. Thus, to improve cataract surgery outcomes, all professionals should attempt to minimize the postoperative complications including binocular vision disorders. To do so requires professionals to perform a comprehensive binocular vision examination protocol that be expected to identify any existing problems. However, this examination requires about 30 min and encompasses a variety of tests to evaluate different aspects of binocular vision, such as visual acuity, refraction, ocular alignment, accommodation, vergence and stereopsis. The specific aim of the study was to determine if a less time-consuming testing protocol would have similar effectiveness compared to a comprehensive examination for the cataract population pre-surgery.

## Methods

### Study design and subjects

The study followed the tenets of the Declaration of Helsinki and the study protocol was approved by the Institutional Review Board of Salus University (HQT1809). The subjects were prospectively and consecutively recruited from the patient population at The Eye Institute of Pennsylvania College of Optometry at Salus University and LewisLASIK—James S. Lewis, MD’s clinic at Elkins Park, Pennsylvania. Eligible subjects were asked to sign a written informed consent and a Health Insurance Portability and Accountability (HIPPA) authorization before any study testing was administered.

Major eligibility criteria included ≥ 50 years old; patient has elected to undergo bilateral cataract extraction with monofocal intraocular lens (IOL) implantation; and best corrected visual acuity no worse than logMAR 0.60 (6/24) in each eye preoperatively. Patients with any ocular pathology other than cataract or strabismus, or a history of previous ocular surgery related to the extraocular muscles, refractive intraocular lens implantation and/or refractive surgery were excluded. Additionally, patients with conditions that impact vision, such as diabetes, systemic muscle diseases, and neurological disorders were also excluded.

### Outcome measurements and diagnostic criteria

The eligibility assessment was performed using routine eye examination procedures to rule out ocular pathology other than cataract and strabismus. A comprehensive battery of binocular vision tests including ocular alignment (unilateral cover test and prism alternate cover test), fusional vergence (step vergence testing), vergence facility (12Δ BO/3Δ BI prism flippers), near point of convergence (near point rule with narrow vertical line) and a symptom survey (Convergence Insufficiency Symptoms Survey, CISS) for binocular vision anomalies was administered to all the subjects by a single, experienced examiner (QQT) before surgery on the first eye, and at the third follow-up visit (approximately 2 months postoperatively) after the surgery on the second eye. All pre- and post-surgical examinations were performed using the habitual prescriptions (glasses that the participants were accustomed to wearing) or trial frames that provided best corrected visual acuity. All participants were presbyopic in this study, so accommodative testing was not performed. A detailed diagnostic classification protocol adapted from Scheiman and Wick [9] was used to identify and classify the presence of a binocular vision anomaly. The detailed protocols for outcome measurements and diagnostic criteria can be found in our previous reports [7, 8].

### Surgical protocol [7]

All cataract surgeries were performed by the same experienced surgeon (JL) under topical anesthesia using the same phacoemulsification system (INFINITI Vision System, Alcon Laboratories, alcon.com). All surgeries were performed with the following protocol: a 2.2-mm superior clear corneal incision followed by continuous circular capsulorhexis, hydrodissection and phacoemulsification cataract extraction. A foldable aspheric monofocal IOL (nanoFLEX, STAAR Surgical Company) was implanted into the capsular bag, followed by a transzonular injection of 0.2 cc Tri-Moxi (Dropless; Harrow Health, harrowinc.com) into the anterior vitreous space to eliminate the need for postoperative medication. The surgical procedure and the implanted IOL were the same for each eye.

### Statistical analysis

All analyses were performed using SPSS Statistics 25.0 with an alpha level of 0.05 to determine the statistical significance. Continuous data were expressed by means and standard deviations (SD), while categorical data were expressed by percent or numeric. A receiver operating characteristic (ROC) curve was plotted to illustrate the diagnostic ability of each test for diagnosing binocular vision anomalies at baseline. The Youden-Index [10] was applied to determine the cut-off points for which (sensitivity + specificity-1) was maximal for each test. For those tests showing a significant diagnostic ability, in addition, ROC curve was plotted for their combinations to seek a more powerful screening protocol. The area under curve (AUC) in ROC curve was used to estimate the performance of each test. An AUC value of 0.5 is considered to suggest no greater than chance, the closer the AUC value is to 1 the better the test. In general, an AUC 0.7 to 0.8 is considered acceptable, 0.8 to 0.9 is considered excellent, and more than 0.9 is considered outstanding [11]. Positive predictive values (PPV) and negative predictive values (NPV) were calculated for each test. For those variables showing a statistical significance in ROC curve, a two-tailed MacNemar’s chi-square test was used to test the statistical significance of difference in the effectiveness of a brief screening protocol and the comprehensive testing battery for determining the risk for binocular vision anomalies. In addition, univariate logistic regression analysis was used to further determine the contribution of each preoperative test to predict pre- and post-surgical binocular vision anomalies.

## Results

### Participant characteristics

Seventy-three participants were included at baseline, 51 of whom completed the post-operative evaluation. Of these 51 participants, the mean age was 70.3 years, and 38 of 51 (74.5%) were female. The mean interoperative interval was 14.06 days, and the mean follow-up time was 57.25 days after the second surgery.

According to the pre-set diagnostic criteria for binocular vision anomalies, 17/51 (33%) of participants were diagnosed with non-strabismic binocular vision anomalies (NSBVAs) at baseline. The most frequent diagnosis was convergence insufficiency 14/51 (27%), one participant had convergence excess, and 2 had basic exophoria. The frequency of NSBVAs decreased to 26% (13/51) after surgery, 11 with convergence insufficiency, 1 with divergence insufficiency, and 1 with fusional vergence dysfunction. No strabismus was detected either pre- or post-surgery. Although no significant change was shown in the NSBVAs frequency pre- and post-surgery, there were a number of participants who converted from a status of NSBVAs to normal binocular vision and vice versa.

### Diagnostic ability of the binocular vision tests

**Fig. 1:**
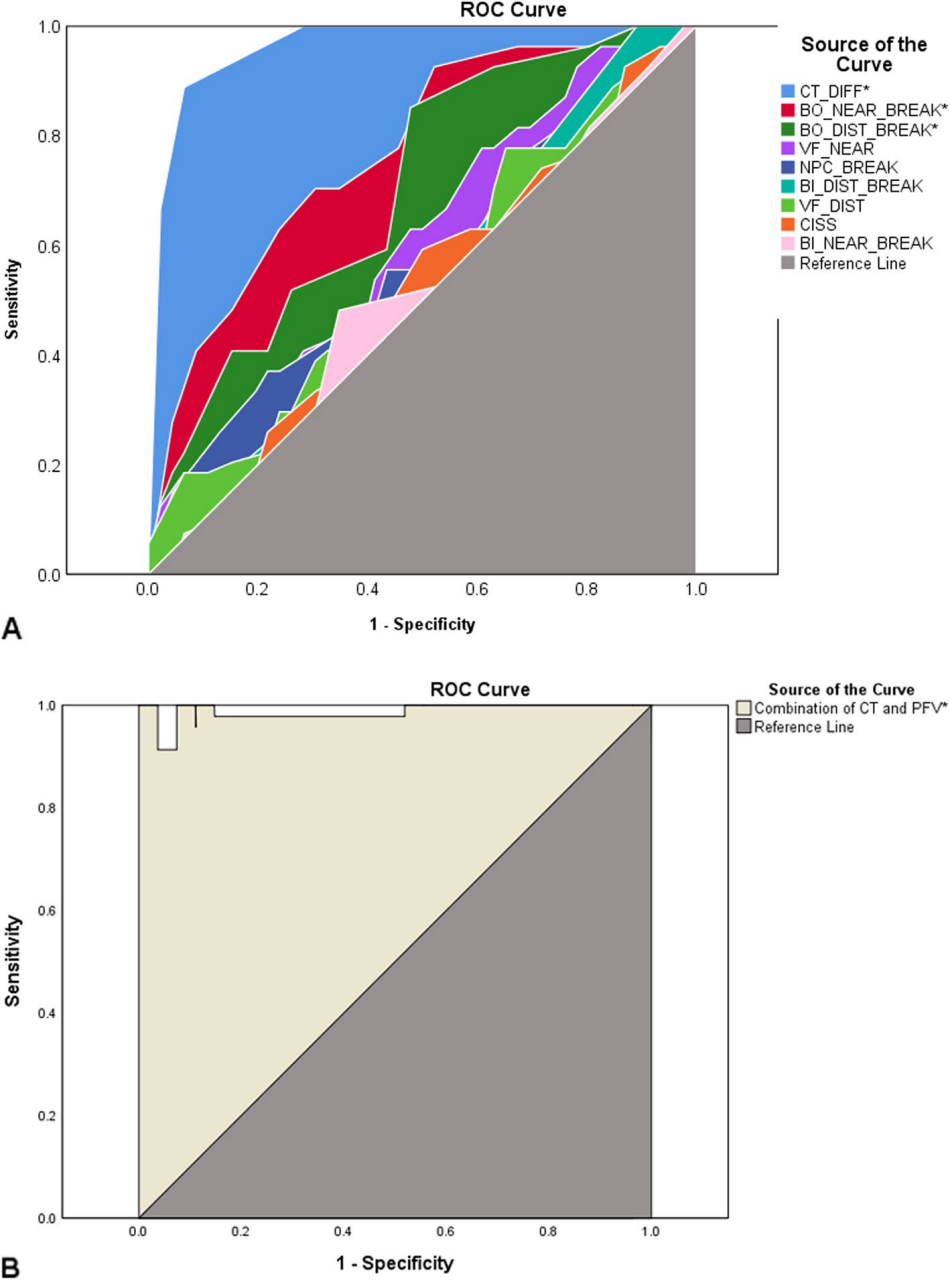
ROC curve showing the diagnostic ability of **A **each binocular vision test, **B **a combination of cover test and positive fusional vergence. (Note: the source of the curve in the figure was sorted by AUC from large to small; CT_DIFF: cover test measured lateral phoria difference between distance and near; BO_NEAR_BREAK: break point of positive fusional vergence at near using base-out prism; BO_DIST_BREAK: break point of positive fusional vergence at distance using base-out prism; BI_DIST_BREAK: break point of negative fusional vergence at distance using base-in prism; BI_NEAR_BREAK: break point of negative fusional vergence at near using base-in prism; VF_NEAR: vergence facility at near; VF_DIST: vergence facility at distance; NPC BREAK: break point of near point of convergence; CISS: convergence insufficiency symptom survey score; CT: cover test; PFV: positive fusional vergence; Reference line: the line with a AUC of 0.5 that indicates a test no better than chance; *: indicating a statistically significant difference from the reference line.)

ROC curve in Fig. 1A illustrates the diagnostic ability of each test. Significant differences were shown for the difference in phoria from distance to near measured by the cover test (*AUC* = 0.96, *P* < 0.01), step vergence testing of positive fusional vergence at distance (*AUC* = 0.71, *P* < 0.01) and near (*AUC* = 0.77, *P* < 0.01), compared to the reference line with AUC of 0.5. The other tests did not show statistically significant differences from the reference line (*P* > 0.05). Further ROC curve was plotted for the combination of difference in distance and near phoria, and positive fusional vergence at distance and near. As shown in Fig. 1B, the combination protocol demonstrated a more robust measure of diagnostic ability than any individual test (*AUC* = 0.98, *P* < 0.01).

The sensitivity, specificity, PPV and NPV were calculated to determine the diagnostic ability of each test and the combination protocol for detecting binocular vision anomalies (Table 1). Using a cut-off point of a 3Δ difference between the distance and near phoria showed an outstanding performance (*sensitivity* = 93.5%, *specificity* = 88.9%, *PPV* = 93.5%, *NPV* = 88.9%). With a cut-off point of 13Δ for positive fusional vergence break at distance demonstrated outstanding performance in sensitivity (85.2%) and PPV (85.7%), with fair performance in specificity (52.2%) and NPV (51.1%); likewise, at the cut-off point of 15Δ in positive fusional vergence break at near demonstrated outstanding performance in sensitivity (92.6%) and PPV (91.7%), whereas fair performance in specificity (47.8%) and NPV (51.0%). This indicates that the test is excellent for identifying binocular vision anomalies but fair for identifying normal binocular vision. However, as a screening test, step vergence testing for positive fusional vergence could still be considered acceptable. The combination of cover test and positive fusional vergence showed a more robust performance than any individual test (*sensitivity* = 91.3%, *specificity* = 100%, *PPV* = 100%, *NPV* = 87.1%). Although the other tests did not show significant differences from the reference in diagnosing binocular vision anomalies, the cut-off values that determined the maximal sensitivity and specificity may be useful for reference in clinical practice in the older presbyopic population. The cut-off points were negative fusional break at distance 7Δ, negative fusional break at near 13Δ, vergence facility at near 11 cycles per minute (CPM), vergence facility at distance 3 CPM, near point of convergence break 7.25 cm, and CISS score 17.5.

**Table 1:** Diagnostic ability of the binocular vision testing

Variable	Cut-off	*AUC*	*Sensitivity*	*Specificity*	*PPV*	*NPV*	*P* value
CT difference between distance and near (Δ)	3	0.96	93.5%	88.9%	93.5%	88.9%	< 0.01*
BI distance break (Δ)	7	0.54	55.6%	45.7%	63.6%	37.5%	0.60
BI near break (Δ)	13	0.50	48.1%	65.2%	68.2%	44.8%	1.00
BO distance break (Δ)	13	0.71	85.2%	52.2%	85.7%	51.1%	< 0.01*
BO near break (Δ)	15	0.77	92.6%	47.8%	91.7%	51.0%	< 0.01*
VF near (CPM)	11	0.61	59.3%	58.7%	71.1%	45.7%	0.13
VF distance (CPM)	3	0.54	74.1%	37.0%	70.8%	40.8%	0.62
NPC break (cm)	7.3	0.58	78.3%	37.0%	67.9%	50.0%	0.28
CISS score	17.5	0.52	59.3%	50.0%	59.0%	32.4%	0.81
Combination protocol (CT + PFV)	−	0.98	91.3%	100.0%	100.0%	87.1%	< 0.01*

Δ: prism diopter; *CPM* Cycle per minute, *AUC* Area under curve, *PPV* Positive predictive value, *NPV* Negative predictive value, *CT* Cover test, *BI* Base-in prism, *BO* Base-out prism, *VF* Vergence facility, *NPC* Near point of convergence, *CISS* Convergence insufficiency symptom survey, *PFV* Positive fusional vergence; *: difference showing statistical significance

To further determine a brief protocol to replace the comprehensive protocol for screening for the risk of binocular vision anomalies, comparisons were performed on the prevalence of binocular vision anomalies determined by the comprehensive protocol and the 3 tests that showed significantly diagnostic effects. As shown in Table 2, binocular vision anomaly prevalence determined by phoria difference with a cut-off point of 3Δ was exactly the same as that by the comprehensive battery (63.0% vs. 63.0%, *P* = 1.00), indicating that cover test alone could be performed as a screening test. On the contrary, solely using distance positive fusional vergence (38.4% vs. 63.0%, *P* < 0.01) or near positive fusional vergence (32.9% vs. 63.0%, *P* < 0.01) significantly underestimated the prevalence of binocular vision anomalies compared to the comprehensive battery, which indicated that neither test should be used alone for screening.

**Table 2:** Brief screening protocol vs. comprehensive testing battery

		**Comprehensive battery**		**Total**	^**¶**^***P***
		**NSBVA**	**NBV**		
➀** Phoria difference between distance and near**	**NSBVA**	43 (58.9%)	3 (4.1%)	46 (63.0%)	1.00
	**NBV**	3 (4.1%)	24 (32.9%)	27 (37.0%)	
	**Total**	46 (63.0%)	27 (37.0%)	73 (100.0%)	
➁** PFV distance break**	**NSBVA**	24 (32.9%)	4 (5.5%)	28 (38.4%)	< 0.01*
	**NBV**	22 (30.1%)	23 (31.5%)	45 (61.6%)	
	**Total**	46 (63.0%)	27 (37.0%)	73 (100.0%)	
➂** PFV near break**	**NSBVA**	22 (30.1%)	2 (2.8%)	24 (32.9%)	< 0.01*
	**NBV**	24 (32.9%)	25 (34.2%)	49 (67.1%)	
	**Total**	46 (63.0)	27 (37.0%)	73 (100.0%)	

①: measured by prism cover test; ②③: measured by step vergence testing; *NSBVA* Non-strabismic binocular vision anomaly, *NBV* Normal binocular vision, *PFV* Positive fusional vergence; ^¶^: 2-tailed McNemar's Chi-Square test; *: difference showing statistical significance

Table 3 illustrates the contribution of each test at baseline to predict a pre- and post-surgical binocular vision disorder using univariate logistic regression analysis. For pre-surgical binocular vision anomaly diagnosis, phoria difference by cover test at distance and near, positive fusional vergence at both distance and near were significant predictors (*P* < 0.05), in which cover test measured phoria difference was the dominant predictor with an odds ratio (OR) of 7.24 (*P* < 0.01). The near vergence facility test demonstrated a borderline significance value (*OR* = 0.95, *P* = 0.05). For post-surgical binocular vision anomaly diagnosis, only the phoria difference showed significant predictive effect (*OR* = 1.25, *P* = 0.02). The near positive fusional vergence showed a borderline significance value (*OR* = 0.96, *P* = 0.05).

**Table 3:** Univariate logistic regression analysis determining the main contributors for predicting the pre- and post-surgical binocular vision anomalies

**Baseline variable**	**Preoperative diagnosis**			**Postoperative diagnosis**		
	***OR***	***95% CI***	***P* value**	***OR***	***95% CI***	***P* value**
CT difference between distance and near	7.24	1.90 to 27.57	< 0.01*	1.25	1.04 to 1.50	0.02*
BI distance break	1.02	0.92 to 1.14	0.67	1.16	0.96 to 1.40	0.12
BI near break	1.01	0.91 to 1.13	0.84	0.99	0.89 to 1.10	0.81
BO distance break	0.93	0.87 to 1.00	0.04*	0.96	0.90 to 1.02	0.16
BO near break	0.92	0.87 to 0.97	< 0.01*	0.96	0.92 to 1.00	0.05
VF near	0.95	0.89 to 1.00	0.05	0.96	0.91 to 1.02	0.17
VF distance	0.98	0.93 to 1.03	0.50	1.00	0.95 to 1.05	0.87
NPC break	1.06	0.89 to 1.25	0.52	1.16	0.96 to 1.41	0.13
CISS score	1.00	0.94 to 1.05	0.89	1.02	0.96 to 1.09	0.45

^*^Difference showing statistical significance; *OR* Odds ratio; *95% CI* 95% confidence interval, *CT* Cover test, *BI* Base-in prism, *BO* Base-out prism, *VF* Vergence facility, *NPC* Near point of convergence, *CISS* Convergence insufficiency symptom survey

## Discussion

A comprehensive battery of binocular vision tests typically takes approximately 30 min and, based on the findings of this study, should be an integral component of the preoperative cataract evaluation. The objective of this study was to ascertain whether a shorter screening process could reasonably identify pre-existing binocular vision issues that may serve as risk factors for postoperative complications.

In a study of children aged 9 to 17, a relevant article, Hussaindeen et al.[12] found that the near point of convergence with penlight and red filter, the difference between distance and near phoria, and monocular accommodative facility were tests with good sensitivity and specificity for the diagnosis of NSBVAs. Our results are comparable to the Hussaindeen et al. data in regard to the difference between distance and near phoria. Compared to Hussaindeen et al., the present study showed larger AUC (0.63 vs. 0.96) with better sensitivity (61.1% vs. 93.5%) and specificity (70% vs. 88.9%) at a larger cut-off value (1.25Δ vs. 3Δ), indicating a more robust effectiveness of difference between distance and near phoria for the diagnosis of NSBVAs in the presbyopic population. Despite the disparity in age groups between the two studies, both underscore the significance of phoria measurements across diverse populations. In addition, in this study the difference between distance and near phoria also showed outstanding PPV and NPV, further supporting the use of this test. Moreover, in this study no difference was found in the prevalence of NSBVAs determined by the comprehensive testing battery and the difference between distance and near phoria, which suggested that solely using this test for screening the risk of NSBVAs may be feasible. The near point of convergence test, which had a significant effect on diagnosis of NSBVAs in Hussaindeen et al.’s study, was not found to have a meaningful effect in the present study. This may be due to the fact that a receded near point of convergence value was found in most subjects in this study, which significantly decreased the specificity of the test. Another test, monocular accommodative facility that was shown to be significant test for diagnosis of non-strabismic binocular vision anomaly was not performed in the present study because of the presbyopic population. However, distance and near positive fusional vergence were significant tests in this study, whereas they were not found to be significant by Hussaindeen et al. Compared to their study, our distance and near positive fusional vergence showed similar AUC (distance: 0.7 vs. 0.71; near: 0.76 vs. 0.77) with higher sensitivity (distance: 80% vs. 85.2%; near: 70% vs. 92.6%) and lower specificity (distance: 60% vs. 52.2%; near: 80% vs. 47.8%) at smaller cut-off points (distance: 15 vs. 13; near: 20 vs. 15). This inconsistency may be due to the significant higher prevalence of convergence insufficiency in the present study (28%) than Hussaindeen et al.’s study (17.6%). Although significant diagnostic effects were shown in distance and near positive fusional vergence, they significantly underestimated the prevalence of NSBVAs when comparing to the comprehensive battery, which precludes using them solely as screening tests for NSBVAs. For a better balance in sensitivity and specificity, a combination of cover test and positive fusional vergence test was analyzed in the present study, in which we found outstanding diagnostic ability (sensitivity = 91.3%, specificity = 100%, PPV = 100%, NPV = 87.1%) for binocular vision disorders in presbyopic population.

Prospective studies on age-related cataract surgery related binocular vision anomalies are scarce. To the best of our knowledge, this is the first study to date to report the contribution of each test at baseline to predict a pre- or post-surgical binocular vision anomaly. The results show that difference between the distance and near phoria and positive fusional vergence break at near are the tests most predictive of postoperative binocular vision anomalies. The odds ratios were 1.25 and 0.96 respectively, indicating that for every 1Δ increase in phoria difference or 1Δ decrease in near positive fusional vergence beak, the odds of postoperative binocular vision anomalies increase by 24.7%, 4% respectively. These tests appear to be more powerful in diagnosing preoperative binocular vision disorders, the odds ratios of phoria difference and near positive fusional vergence were 7.24 (*P* < 0.01) and 0.92 (*P* < 0.01) respectively. Moreover, there were more tests showing significance in diagnosing preoperative binocular vision disorders though with borderline significance values, including distance positive fusional vergence (*OR* = 0.93, *P* = 0.04) and near vergence facility (*OR* = 0.95, *P* = 0.05). This result further supports the robustness of the cover test and positive fusional vergence in screening for the binocular vision anomalies, and also confirms their significance in predicting a postoperative condition. We recommend that the cataract surgeons perform at least a cover test at both near and distance, or a combination with a near positive fusion vergence test to help predict potential post-surgical binocular vision complications.

This study has three limitations: Firstly, there was a relatively high dropout rate observed among the participants with 22 out of 73 individuals (30%) discontinuing their involvement in response to health or transportation challenges encountered by some elderly subjects during follow-up assessments. In order to mitigate this issue, we performed a sensitivity analysis under an assumption that all dropouts had developed a binocular vision anomaly. The outcomes revealed no significant difference in terms of prevalence for binocular vision anomalies before and after surgery (33% vs. 49%, McNemar's test, *P* = 0.08), thereby indicating that the elevated dropout rate did not appear to introduce any bias into our findings; Secondly, it should be noted that generalizing these results beyond older adults with cataracts alone might be limited in scope; however, given the substantial occurrence of cataracts within this demographic group, these research findings still offer valuable insights into understanding binocular vision among such individuals. Lastly, the investigation of cataract type and visual acuity's impact on ocular alignment was not conducted, leaving room for future studies to address this aspect.

In conclusion, distance and near phoria difference measured by cover test has similar effectiveness as a comprehensive binocular vision testing protocol for the diagnosis of binocular vision anomalies. It can be used as screening test for binocular vision anomalies before cataract surgery. Distance and near positive fusional vergence measured by step vergence testing also have significant predictive value, however, when used as standalone tests, they may underestimate the prevalence of binocular vision anomalies. A combination of the cover test and positive fusional vergence test is an outstanding screening protocol for binocular vision anomalies before cataract surgery. A brief screening protocol can be as effective as a comprehensive binocular vision examination protocol in identifying cataract patients with binocular vision anomalies.

## Supplementary Information

Supplementary Material 1.

## Data Availability

The datasets used during the current study are available from the corresponding author on reasonable request.

## Abbreviations

ROC: Receiver operating characteristic
AUC: Area under curve
IOL: Intraocular lens
CISS: Convergence insufficiency symptoms survey
PPV: Positive predictive values
NPV: Negative predictive values
NSBVAs: Non-strabismic binocular vision anomalies
HIPPA: Health Insurance Portability and Accountability
CPM: Cycles per minute
CT: Cover test
PFV: Positive fusional vergence
BI: Base-in prism
BO: Base-out prism
VF: Vergence facility
NPC: Near point of convergence
OR: Odds ratio
95% CI: 95% Confidence interval
