# Supplementary material for: A brief effective screening protocol for identifying cataract patients with binocular vision anomalies
Source: BMC Ophthalmol. 2024 Dec 18;24:536. doi: 10.1186/s12886-024-03807-w (PMC11657689; doi:10.1186/s12886-024-03807-w)
Supplement: Supplementary file 1 — Supplementary Material 1. [file 12886_2024_3807_MOESM1_ESM.docx]

**Setup and procedure of the comprehensive examination protocol for**

**binocular vision**

- **COVER TEST (including unilateral and alternate cover test)**

**Equipment needed**

1. Gulden fixation stick #15302 with 40 cm string attached to fixation stick
2. Vision chart for distance
3. Occluder
4. Gulden B-16 prism bar (1, 2, 4, 6, 8, 10, 12, 14, 16, 18, 20, 25, 30, 35, 40, and 45 prism diopters)

**Setup**

1. Use habitual glasses or trial lenses for both distance and near test.
2. Isolate a single letter two lines above threshold of acuity for distance.
3. Hold Gulden fixation stick 40 cm from the participant and direct the his/her attention to

the isolated letter.

1. Set the examination room well-illuminated.
2. Ensure that prisms are clean and that there are no scratches that may interfere with the participant’s ability to see the target.

**Procedure**

1. Instruct the participant to fixate the distance letter and to “keep it clear” throughout testing.
2. Cover the participant’s right eye and watch left eye as right eye is covered.
3. Cover the participant’s left eye and watch right eye as left eye is covered.
4. Repeat the unilateral cover test several times to determine if strabismus is present.
5. Allow the participant adequate time to regain fixation.
6. Record whether strabismus is present. If a strabismus is present, record whether it is

intermittent or constant.

1. Neutralize the alternate cover test according to the following procedure:
2. Introduce prism with the appropriate direction of base.
3. Cover one eye with the occluder, interposing the prism behind the occluder.
4. Switch the occluder and observe the eye movement behind the prism.
5. Increase the magnitude of prism until no eye movement is observed.
6. Continue adding prism until the first reversal.
7. Record the amount and base of prism that resulted neutrality before the first reversal.
8. Repeat the same procedure for near.
9. During the test the examiner should also observe whether a vertical deviation is present. If a vertical deviation is present, the examiner should measure the magnitude of the deviation and record.

- **STEP VERGENCE TESTING**

**Equipment needed**

1. Gulden B-16 horizontal prism bar (1, 2, 4, 6, 8, 10, 12, 14, 16, 18, 20, 25, 30, 35, 40, and 45 prism diopters)
2. Gulden fixation stick #15302 with 40 cm string attached to fixation stick

**Setup**

1. Use habitual glasses or trial lenses for both distance and near test.
2. Isolate a single letter two lines above threshold of acuity for distance.
3. Hold Gulden fixation stick 40 cm from the participant and direct the his/her attention to

the isolated letter.

1. Set the examination room well-illuminated.
2. Ensure that prisms are clean and that there are no scratches that may interfere with the participant’s ability to see the target.

**Procedure**

1. Negative fusional vergence at distance:
2. Instruct the participant to fixate the distance letter and to “keep it clear” throughout testing.
3. Place the horizontal prism bar with the flat side towards the participant in a base-in orientation with the participant viewing through 1∆ BI.
4. Ask the participant to “tell me when the letters become blurred or become double, but try to keep the target single as long as possible”.
5. Increase magnitude of BI prism at 2∆ per second, pausing at each prism to confirm that the target is “single and clear”.
6. If the participant reports blur, pause and note BI prism amount then continue to increase BI prism pausing at each prism to confirm that the target is “single.” When the participant reports double, ask the participant “Does it stay two or does it come back into one?” Continue to introduce BI prism if the participant recovers single vision. When the participant can no longer maintain single vision, and has diplopia, note the BI prism amount and record this value at the “BI break”.
7. After the participant reports diplopia, increase the BI prism by 5∆, and then reduce the BI prism at a rate of 2∆ per second until the participant reports single vision. Consider this the “recovery” finding. If recovery finding is higher than the break, repeat the entire measurement (blur, break and recovery). Accurately record blur, break and recovery findings.
8. If diplopia is not reported but examiner notes loss of fusion, the prism through which fusion is lost should be recorded as the “break” finding. Likewise, an examiner observation of recovery of fusion should be recorded as “recovery”.
9. If the participant is able to fuse the largest (45∆) prism, record 50∆ for the break value and have the participant close or cover one eye to break fusion so that recovery can be measured. Record the amount of prism through which the participant was able to regain fusion (maximum value would be 45∆).
10. Repeat the same procedure for negative fusional vergence at near by replacing the distance target with a Gulden fixation stick.
11. At least 30 seconds after the negative fusional vergence testing, repeat the procedure for positive fusional vergence at distance and near by pacing the prism bar in a base-out orientation.

- **VERGENCE FACILITY TESTING**

**Equipment needed**

1. Gulden vergence facility prism (12 base-out/3 base-in flipper)
2. Gulden fixation stick #15302 with 40 cm string attached to fixation stick
3. Stopwatch

**Setup**

1. Use habitual glasses or trial lenses for both distance and near test.
2. Isolate a single letter two lines above threshold of acuity for distance.
3. Hold Gulden fixation stick 40 cm from the participant and direct the his/her attention to

the isolated letter.

1. Set the examination room well-illuminated.
2. Ensure that prisms are clean and that there are no scratches that may interfere with the participant’s ability to see the target.

**Procedure**

1. Instruct the participant to fixate the distance letter and ensure the letter is single and clear.
2. Place 12 BO side of the flipper before one of the participant’s eyes. Ask him/her to try to make letter single and clear as quickly as possible.
3. Instruct the participant to say “single” as soon as the letter is single and clear.
4. When letter is reported to be single and clear, quickly flip the flipper to the 3 BI side, again instructing the participant to report when letter is single and clear.
5. Prepare to begin timing for one minute using a stopwatch.
6. Start timing while placing the 12 BO prism in front of the participant’s eye. Continue to alternate sides of prism flipper for 1 minute, while counting the number of “flips” of the prism that the participant was able to make single and clear.
7. Record number of prism flips and presentation with most difficulty (BI more difficult, BO more difficult, equally difficult) on data collection form.

- **NEAR POINT OF CONVERGENCE**

**Equipment needed**

1. A thin vertical line target mounted on a Bernell Accommodation Convergence rule

**Setup**

1. Use habitual glasses or trial lenses for near optical correction.
2. Make sure ambient and overhead lighting provide good illumination.

**Procedure**

1. Hold the edge of rule on the center of the participant’s forehead just above the level of his/her brow. Begin with the vertical line target placed at the 40-cm mark on the rule.
2. Instruct the participant to “look at the target and report when it becomes double or break into two but try to keep the target one/single as long as possible”. Slowly (1-2 cm/sec) move target toward the participant. When diplopia is reported, stop moving the target and ask the participant “Does it stay two or does it come back into one?”.
3. If it comes back into one within 1-2 seconds, continue slowly moving the target towards the participant until he/she is unable to regain fusion. Do not hold the target in place for longer than 2 seconds.
4. If it stays double, this endpoint is the near point of convergence break.
5. If the examiner observes a loss of fusion (without a report of double), the point at which the examiner observed a loss of fusion is considered the near point of convergence break.
6. If the participant did not break, have the participant close or cover one eye for 3-5 seconds to break fusion so that recovery can be measured.
7. Ask the participant to tell you “When it comes back together into one” and slowly move the target away from the participant until the he/she reports single vision or the examiner observes a recovery of fusion. This is the near point of convergence recovery.
8. Record the near point of convergence break and recovery values.
9. Measure the break and recovery as described above three times, waiting 10 seconds between paired break/recovery measurements.

- **CONVERGENCE INSUFFICIENCY SYMPTOM SURVEY (CISS)**

**Material needed**

CISS questionnaire (as follow)


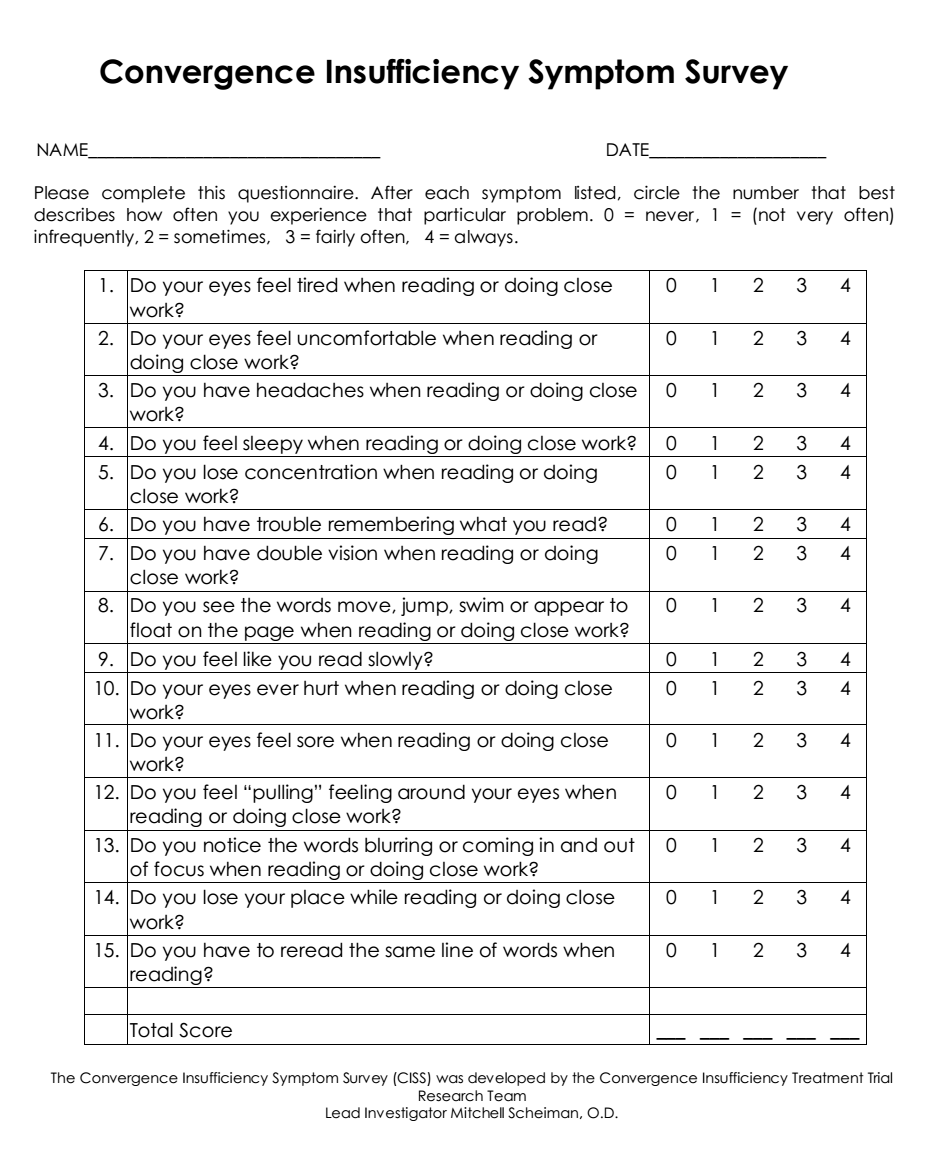


**Setup**

1. Use habitual glasses or trial lenses for near optical correction.
2. Make sure ambient and overhead lighting provide good illumination.

**Procedure**

1. Fill in the demographic information for each participant on the top of the survey.
2. Instruct the participant as follows: “Please complete this questionnaire. After each symptom listed, circle the number that best describes how often you experience that particular problem. 0 = never, 1 = (not very often) infrequently, 2 = sometimes, 3 = fairly often, 4 = always.”
3. Sum the total CISS score and record it at the bottom of the survey.
